# Two Cases of Plasmablastic Myeloma Mimicking Plasmablastic Lymphoma With In‐Depth Review of Literature

**DOI:** 10.1002/cnr2.70094

**Published:** 2025-02-05

**Authors:** Sakiko Kitamura, Kazuho Morichika, Sawako Nakachi, Taeko Hanashiro, Riko Miyagi, Tomo Nakajima, Yukiko Nishi, Keita Tamaki, Takuya Fukushima, Hiroaki Masuzaki

**Affiliations:** ^1^ Division of Endocrinology, Diabetes and Metabolism, Hematology and Rheumatology, Second Department of Internal Medicine, Graduate School of Medicine University of the Ryukyus Okinawa Japan; ^2^ Laboratory of Immunohematology, School of Health Sciences, Faculty of Medicine University of the Ryukyus Okinawa Japan

**Keywords:** Ki‐67, plasmablastic lymphoma (PBL), plasmablastic myeloma (PBM)

## Abstract

**Background:**

Plasmablastic myeloma (PBM) is a rare, aggressive subtype of multiple myeloma (MM) with poor prognosis. On the other hand, plasmablastic lymphoma (PBL) is an aggressive B‐cell lymphoma with a plasmacytic phenotype. Importantly, PBM is difficult to distinguish from PBL, because clinical features of both diseases closely overlap. We report two cases of PBM accompanied by apparent extramedullary lesions.

**Case:**

Case 1: A 38‐year‐old female complained of fatigue. She presented with pancytopenia, splenomegaly, a soft tissue lesion over the chest wall, and multiple osteolytic lesions. Initially, pathology of the soft tissue established a diagnosis of PBL. She received two cycles of EPOCH, leading to considerable improvement. She then received daratumumab (Dara) and lenalidomide, achieving remission for two years. Case 2: A 60‐year‐old male was evaluated for multiple tumors of the pancreas and retroperitoneum. A biopsy of the pancreatic tumor identified plasmacytoid cells, whereas a biopsy of the bone marrow showed no evidence of plasma cells. Therefore, he was initially diagnosed as having multiple plasmacytomas and received 3 cycles of chemotherapy with bortezomib (Bor), lenalidomide, and dexamethasone, but in vain. Once Bor was replaced to Dara, he rapidly developed panperitonitis and ascites filled with plasmablasts and eventually died of multiple organ failure.

**Conclusion:**

As there is no standard treatment for PBM, our cases raise a possibility that combination therapy with anti‐myeloma and anti‐lymphoma regimens may provide better outcomes. In addition, the Ki‐67 proliferation index would be a useful tool to diagnose PBM.

## 1 Introduction

Multiple myeloma (MM) is an age‐related indolent disorder of plasma cells. In particular, plasmablastic myeloma (PBM) is an extremely rare subtype of MM in which higher than 2 percent of the clonal plasma cells represent plasmablastic morphology with poor prognosis and survival [1, 2]. On the other hand, plasmablastic lymphoma (PBL) is an aggressive B‐cell lymphoma representing a plasma cell phenotype [3]. Prototypical PBL is associated with infection of human immunodeficiency virus (HIV) and also a frequent co‐infection with Epstein–Barr virus (EBV) [4]. In some cases, differential diagnosis between PBM and PBL is difficult because the morphology and immunophenotype closely overlap between the two diseases [5]. Generally, PBM has been treated with anti‐myeloma chemotherapy composed of proteasome inhibitors, immunomodulatory agents, alkylators, recombinant monoclonal antibodies to CD38, and autologous stem cell transplantation [6, 7, 8, 9]. On the other hand, combined chemotherapy with anti‐lymphoma drugs has been recommended for PBL [10].

We here report two extremely rare cases of plasmablastic myeloma mimicking plasmablastic lymphoma, with an extensive review of literature. These cases were treated between March 2021 and June 2023 in our institute. The Case 1 received anti‐lymphoma chemotherapy promptly followed by anti‐myeloma chemotherapy, resulting in a favorable outcome. In contrast, Case 2 showed resistant against anti‐myeloma chemotherapy and transformation into aggressive type during the clinical course.

## 2 Case

**FIGURE 1:**
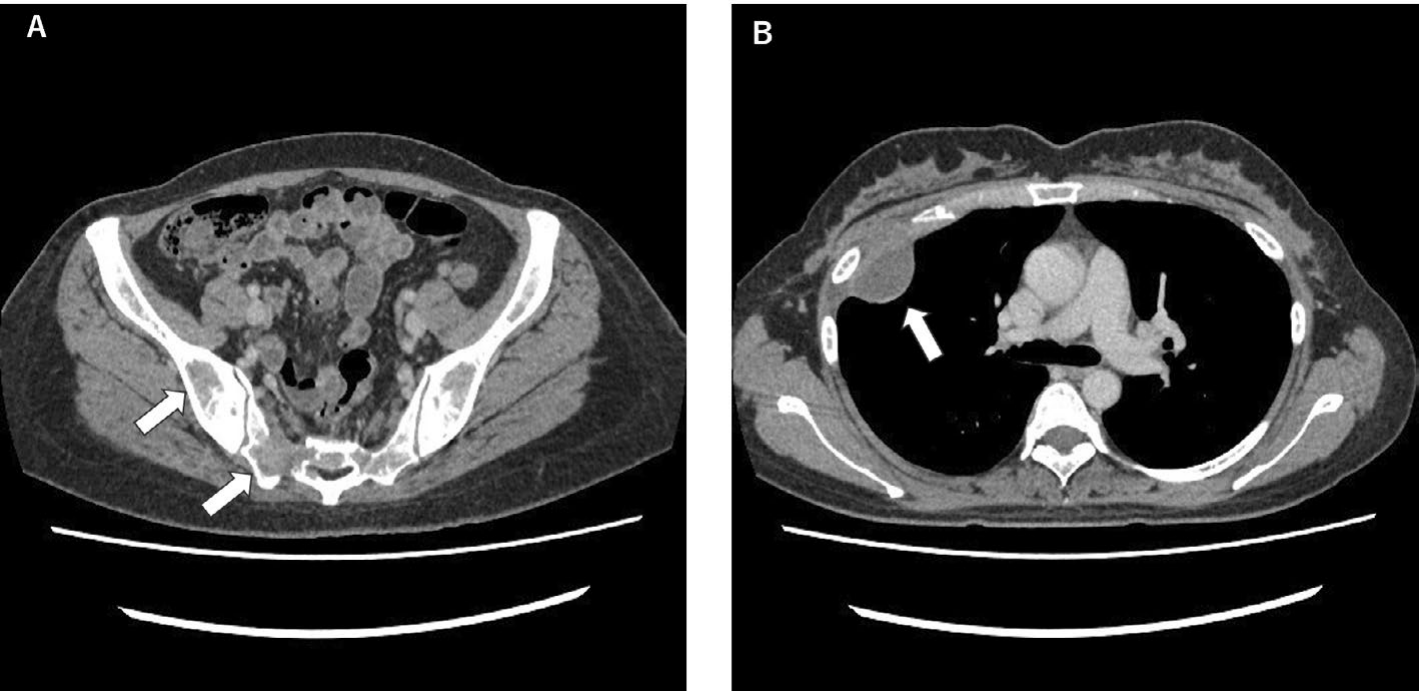
Abdominal and Chest Computerized Tomography Imagings (Case 1). An abdominal CT imaging of Case 1 showed multiple osteolytic lesions involving pelvic bones (Panel A, indicated by arrows). A Chest CT imaging showed a soft‐tissue mass developed over the right chest wall (Panel B, indicated by an arrow).

**FIGURE 2:**
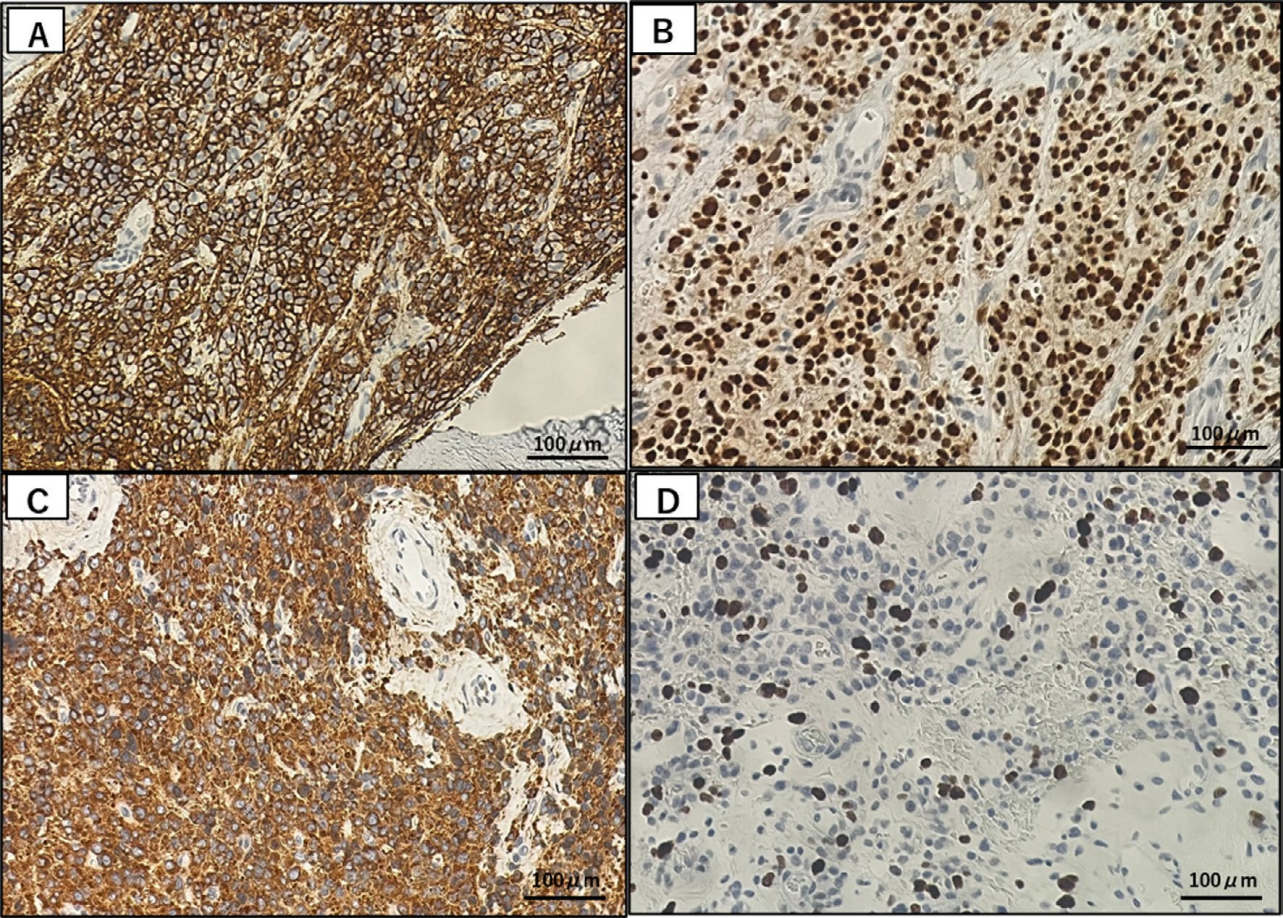
Histopathological analyses of the soft‐tissue mass over the chest wall (Case 1). These abnormal cells were positive for CD138 which is a marker of plasmacytic differentiation (A), Multiple myeloma oncogene 1 (MUM1) which is normally expressed in terminal stages of differentiation of B‐cells in the germinal center and plasma cells (B), and λ‐chain (C). These cells showed 30% of the Ki‐67 index (D). A line of findings above was in agreement with the diagnosis of PBM.

*Case 1*: A 38‐year‐old female complained of fatigue evolving for several months. The patient showed no palpable surface lymph nodes. Physical examination identified marked splenomegaly and tender pain in the section of the lumbar vertebrae. Laboratory tests showed severe anemia and pancytopenia (white blood cell count 3500 cells/μL, hemoglobin level 2.9 g/dL, platelet count 79 × 10^3^/μL). Blood chemistry studies revealed that immunoglobulin (Ig) levels were markedly decreased: IgA at 7 mg/dL, IgG at 208 mg/dL, and IgM at 4 mg/dL. Notably, Bence‐Jones lambda (λ) monoclonal protein was detected. In addition, the test for HIV was negative. Results of other laboratory tests are shown in Table 1. Computer tomography (CT) imaging showed multiple osteolytic lesions involving the mandible, sternum, ribs, vertebrae, and pelvic bones, as well as a soft‐tissue mass developed over the right chest wall (Figure 1). Bone marrow (BM) biopsy demonstrated diffuse myelofibrosis with plasmacytoid neoplastic cell infiltration; however, neither JAK2 nor CALR gene mutation was detected by allele‐specific PCR amplification. Abnormal cells of BM were positive for cluster of differentiation (CD) markers such as CD138 and λ‐chains by immunohistochemical staining. A Biopsy of the soft‐tissue mass over the chest wall demonstrated plasmacytoid cells positive for CD138, Bcl2, λ‐chain, and MUM1, but negative for CD3, CD10, CD30, CD79a, kappa (κ)‐chain, Bcl6, PAX5, and in situ hybridization for EBV‐encoded RNA (EBER), with moderate Ki‐67 staining of 30% of the cells (Figure 2). Based on the aforementioned findings atypical for MM, including the onset age and phenotype of myelofibrosis with splenomegaly, she was initially diagnosed as PBL. She received two cycles of chemotherapy with EPOCH (etoposide, prednisone, vincristine, cyclophosphamide, doxorubicin), leading to considerable improvement. Reviewing her clinical features, she was re‐diagnosed as PBM based on the moderate Ki‐67 index and negativity of EBER. She was treated with DLd (daratumumab, lenalidomide, and dexamethasone), resulting in remission. The intrathoracic mass disappeared, and her serum λ‐light chain decreased from 4262 to 10 mg/L. She has continued DLd without recurrence for two years.

**TABLE 1:** Laboratory data on admission.

Variable	Reference range	Case 1	Case 2
Hemoglobin (g/dL)	13.7–16.8	2.9	12.7
Hematocrit (%)	40.7–50.1	9.6	37.4
White blood cell count (per μL)	3300–8600	3500	4000
Differential count (%)			
Neutrophils	40.7–77.0	66.5	46.1
Lymphocytes	16.0–49.5	28.0	43.5
Monocytes	2.0–10.0	4.0	9.5
Eosinophils	0.0–8.5	0.5	0.7
Platelet count (× 10^3^ per μL)	158–348	79	205
Urea nitrogen (mg/dL)	8.0–20.0	7.2	12
Creatinine (mg/dL)	0.65–1.07	0.57	1.0
Total bilirubin (mg/dL)	0.4–1.5	4.2	0.5
Alanine aminotransferase (U/L)	10–42	11	26
Aspartate aminotransferase (U/L)	13–30	11	21
Alkaline phosphatase (U/L)	38–113	NA	68
Amylase (U/L)	44–132	NA	76
Lactate dehydrogenase (U/L)	124–222	120	235
Urate (mg/dL)	3.7–7.8	6.0	5.3
Total protein (g/dL)	6.6–8.1	5.1	8.0
Albumin (g/dL)	4.1–5.1	3.8	3.3
C‐reactive protein (mg/L)	0.0–0.14	0.01	0.27
Immunoglobulin G (mg/dL)	861–1747	208	2659
Immunoglobulin M (mg/dL)	33–183	4	22
Immunoglobulin A (mg/dL)	93–393	7	78
Free light κ‐chain (mg/L)	3.3–19.4	0.6	115
Free light λ‐chain (mg/L)	5.7–26.3	4262	13.1
κ/λ ratio	0.26–1.65	< 0.01	8.77
Soluble interleukin‐2 receptor (U/mL)	121–613	2133	437

**FIGURE 3:**
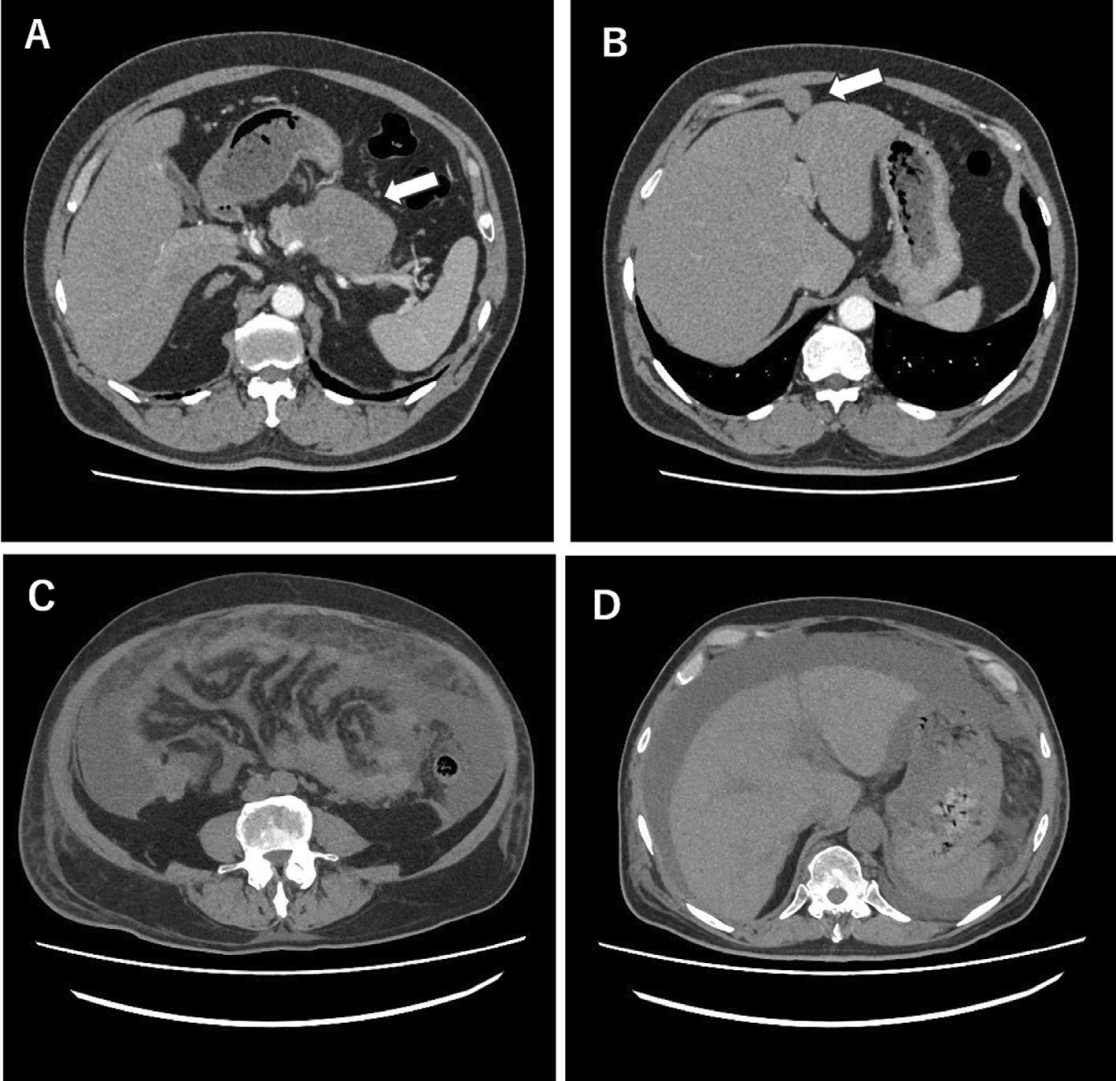
Abdominal computerized tomography imagings at the first presentation and plasmablastic transformation (Case 2). An abdominal CT imaging of obtained at the first presentation of Case 2 showed a noninvasive mass in the head of the pancreas (Panel A, indicated by an arrow) and multiple masses in the peritoneum (Panel B, indicated by an arrow). An abdominal CT imaging of obtained after local irradiation therapy for the head of the pancreas of Case 2 showed pan‐peritonitis (Panel C) and massive ascites (Panel D).

**FIGURE 4:**
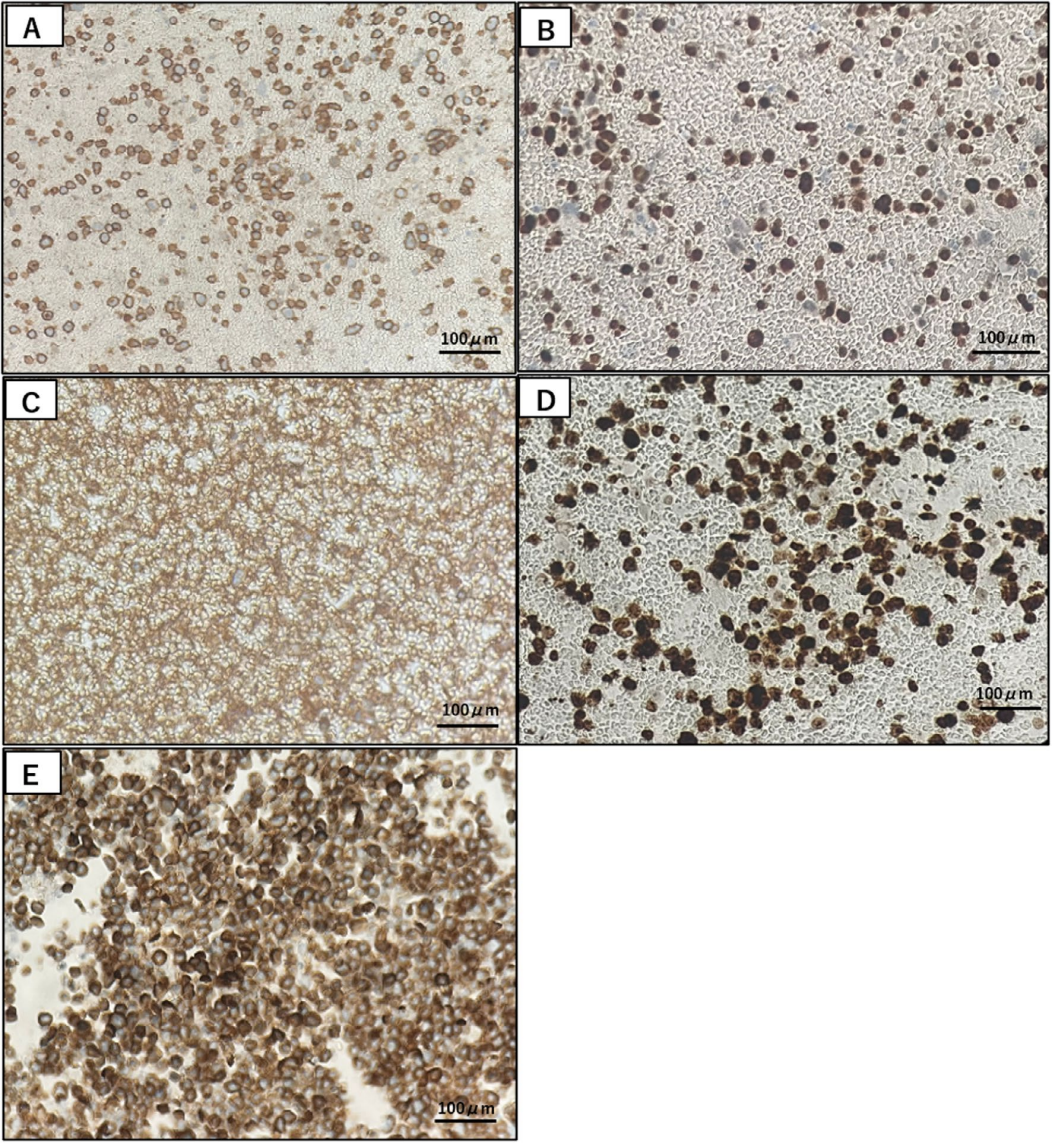
Histopathological analyses of the pancreatic tumor at the first presentation and the ascites at the plasmablastic transformation (Case 2). Histopathological findings of the pancreatic tumor of Case 2 (Panel A–D). Abnormal cells were positive for CD138 which is a marker of plasmacytic differentiation (A), Multiple myeloma oncogene 1 (MUM1) which is normally expressed in terminal stages of differentiation of B‐cells in the germinal center and plasma cells (B), and κ‐chain (C). These cells showed 50% of the Ki‐67 index (D). A line of findings was in agreement with the diagnosis of PBM. Histopathological analysis of the ascites of Case 2 showed infiltration by plasma cells with plasmablastic features, diffusely positive for CD138 (Panel E). This finding suggests that the patient developed pan‐peritonitis caused by a rapid progression of PBM.

*Case 2*: A 60‐year‐old male was evaluated for a tumor of the pancreas and chronic kidney disease with proteinuria. Blood chemistry studies demonstrated an elevation of IgG at 2659 mg/dL. In contrast, low levels of IgA at 78 mg/dL and IgM at 22 mg/dL were noted. Results of other laboratory tests are shown in Table 1. CT imaging showed a noninvasive mass in the head of the pancreas and multiple masses in the peritoneum (Figure 3). Renal biopsy showed a typical pathology of myeloma kidney. Endoscopic biopsy of pancreatic tumor identified plasmacytoid cells. Immunohistochemically, the abnormal cells were positive for CD38, CD138, MUM1, Bcl2, and κ‐chain but negative for CD30, CD79a, λ‐chain, Bcl6, PAX5, and EBER, with moderate Ki‐67 staining of 50% of the cells (Figure 4). Because a biopsy of bone marrow showed no evidence of plasma cells, he was diagnosed with multiple myeloma initially. He received 3 cycles of chemotherapy with VRd (bortezomib, lenalidomide, dexamethasone), but in vain, and treatment was stopped because he developed pneumonia and severe peripheral neuropathy. During the drug withdrawal period, the tumors enlarged steadily. Although he received chemotherapy with DLd followed by local irradiation therapy, he rapidly developed panperitonitis and massive ascites filled with plasmablasts Figures 3 and 4. The findings of abnormal cells with plasmablastic morphology led to a re‐diagnosis of PBM, and he received carfilzomib but died of multiple organ failure due to tumor lysis syndrome.

## 3 Discussion

We report two cases of plasmablastic myeloma (PBM) closely mimicking PBL. PBM is a blastic morphological variant of plasma cell neoplasm. PBM and PBL are known to share similar clinical and pathological features with poor prognosis [1, 2]. In particular, PBM showing extramedullary involvement and accumulation of body cavity fluid as initial symptoms is difficult to distinguish from PBL [5].

Chen et al. reported retrospective cohort analyses of 25 patients with PBM diagnosed between 2001 and 2021 [11]. In the report, fifteen patients presented extramedullary involvement. Among the patients, soft tissue and/or skin regions were the most common (*n* = 10, 67%), followed by pleural effusion (*n* = 7, 47%), the lung (*n* = 5, 33%), lymph node (*n* = 3; 20%) [11]. In our patients, Case 1 showed multiple osteolytic lesions and soft‐tissue mass developed over the chest wall, and Case 2 showed masses in the head of the pancreas and peritoneum. Such a manifestation would make it difficult to diagnose as PBM. Generally, PBM has been treated with combinations of steroids, proteasome inhibitors, immunomodulatory agents, alkylators, recombinant monoclonal antibodies to CD38, and autologous stem cell transplantation [6, 7, 8, 9]. In Chen's reports, among the 25 patients with PBM, twenty‐one patients received chemotherapy or concurrent chemoradiotherapy. In the cohort, nineteen patients (79%) died of disease, four patients (17%) were alive with disease, and one patient (4%) who had received autologous stem cell transplantation was alive with remission. Taken together, the 1‐, 2‐, and 5‐year OS rates were 71%, 52%, and 10%, respectively [11]. Case 1 was successfully treated with EPOCH followed by DLd and achieved complete remission for more than two years without autologous stem cell transplantation. PBM is sometimes diagnosed as a *de novo* case but also identified in plasmablastic transformation from extramedullary MM or plasmacytoma [12]. Liu et al. reported 10 cases of extramedullary MM developing plasmablastic transformation [13]. The treatment regimens after the transformation included bortezomib and dexamethasone in addition to thalidomide, elotuzumab, PACE (cisplatin, doxorubicin, cyclophosphamide, etoposide), or EPOCH with a median survival of 4.5 months (range: 2 to 31 months) [13]. The Case 2 received only anti‐myeloma regimens, which were ineffective, and died 2 months after transformation. To date, PBL is an aggressive B‐cell lymphoma with no standard care; however, combined chemotherapy composed of anti‐lymphoma drugs has been recommended for PBL [10]. The regimens consist of CHOP (cyclophosphamide, doxorubicin, vincristine, and prednisone) or CHOP‐like regimens, hyper‐CVAD‐MA (hyper‐fractionated cyclophosphamide, vincristine, doxorubicin, dexamethasone, and high‐dose methotrexate and cytarabine), CODOX‐M/IVAC (cyclophosphamide, vincristine, doxorubicin, high‐dose methotrexate/ifosfamide, etoposide, and high‐dose cytarabine), and EPOCH [3]. Regrettably, there has been no gold standard of diagnostic algorithm for both PBM and PBL. Furthermore, considerable difficulty in accurate diagnosis between two diseases cause dilemma of choosing therapy [14]. Clinically, PBM can be distinguished from PBL in some cases by the presence of hypercalcemia, renal insufficiency, anemia, and osteolytic lesions, but borderline cases are treated with V‐EPOCH (bortezomib plus EPOCH), which consists of an anti‐myeloma drug plus an anti‐lymphoma regimen [15, 16].

So far, some diagnostic algorithms to differentiate PBM and PBL have been proposed [14, 17]. In Ahn's diagnostic algorithm, patients are initially categorized by the positivity of EBER. At this time point, signs of myeloma are not considered, and patients with positivity of EBER are classified as PBL [14]. In this context, both of our cases were classified as PBM by Ahn's algorithm. However, this algorithm seems to be inadequate for diagnosis of PBL, because the rate of EBER positivity is at most 70% [18]. In Chen's diagnostic algorithm, signs of myeloma are considered. The finding of positivity of EBER is not directly linked to diagnosis as PBL [17]. As Chen's algorithm is based on typical myeloma signs and sites of onset, Case 1 does not reach the differential diagnosis. Actually, Case 1 did not show typical signs of myeloma. She represented myelofibrosis with splenomegaly and an uncommon age of onset for typical MM. Alternatively, the use of Ki‐67 to diagnose PBM was reported [19]. Antigen Ki‐67, also known as Ki‐67 or Marker of Proliferation Ki‐67 (MKI67), is a protein in humans encoded by the MKI67 gene, Ki‐67 is often considered a prognostic biomarker in certain malignancies [20]. Previous reports demonstrated that patients with negativity of EBER were diagnosed with PBM when they had lower than 80% of Ki‐67 index, regardless of signs of myeloma [19]. In addition, Ki‐67 is low in typical MM (3%–15%) [21, 22] and significantly higher in PBL (83%–90%) [3]. Therefore, Ki‐67 is one of the useful clues to diagnose PBM. In this content, both of our cases showed negative for EBER and moderate Ki‐67 proliferation index (30% of Case 1, 50% of Case 2). The Case 2 showed an apparent transformation into the aggressive type. However, Ki‐67 at the time of initial diagnosis was slightly elevated, and in case PBM had been diagnosed earlier, more intensive chemotherapy might have been administered.

In summary, we report two cases of PBM mimicking of PBL. Our findings highlight that the treatment consisting of anti‐myeloma drugs combined with anti‐lymphoma regimens for PBM may provide better outcomes. Moreover, a moderate Ki‐67 proliferation index would be a useful tool to accurately diagnose PBM. Further studies are warranted about the criteria for diagnosis and therapeutic approach in PBM.

## Author Contributions

**Sakiko Kitamura:** conceptualization, data curation, formal analysis, investigation, methodology, project administration, resources, writing – original draft. **Kazuho Morichika:** investigation, writing – review and editing, data curation. **Sawako Nakachi:** supervision, project administration, investigation, writing – review and editing. **Taeko Hanashiro:** conceptualization, data curation, resources, writing – review and editing. **Riko Miyagi:** writing – review and editing. **Tomo Nakajima:** writing – review and editing. **Yukiko Nishi:** resources, data curation, writing – review and editing. **Keita Tamaki:** writing – review and editing. **Takuya Fukushima:** writing – review and editing. **Hiroaki Masuzaki:** supervision, writing – review and editing.

## Ethics Statement

This study was approved by the ethics committee of the University of the Ryukyus (No. 23‐2169‐00‐00‐00).

## Consent

The patients and their family wrote an informed consent to publish this report.

## Conflicts of Interest

The authors declare no conflicts of interest.

## Data Availability

The data that support the findings of this study are available from the corresponding author upon reasonable request.
